# Allelic diversity in the transcriptomes of contrasting rust-infected genotypes of *Lathyrus sativus*, a lasting resource for smart breeding

**DOI:** 10.1186/s12870-014-0376-2

**Published:** 2014-12-19

**Authors:** Nuno Felipe Almeida, Susana Trindade Leitão, Nicolas Krezdorn, Björn Rotter, Peter Winter, Diego Rubiales, Maria Carlota Vaz Patto

**Affiliations:** Instituto de Tecnologia Química e Biológica António Xavier, Universidade Nova de Lisboa, Av. da República, 2780-157 Oeiras, Portugal; GenXPro GmbH, D-60438 Frankfurt am Main, Germany; Institute for Sustainable Agriculture, CSIC, E-14080 Córdoba, Spain

**Keywords:** Grass pea, Partial resistance, Pathogen effectors, RNA-seq, SNP, *Uromyces*

## Abstract

**Background:**

Grass pea (*Lathyrus sativus* L.) is a valuable resource for potentially durable partial resistance to rust. To gain insight into the resistance mechanism and identify potential resistance genes, we generated the first comprehensive transcriptome assemblies from control and *Uromyces pisi* inoculated leafs of a susceptible and a partially rust-resistant grass pea genotype by RNA-seq.

**Results:**

134,914 contigs, shared by both libraries, were used to analyse their differential expression in response to rust infection. Functional annotation grouped 60.4% of the contigs present in plant databases (37.8% of total) to 33 main functional categories, being “protein”, “RNA”, “signalling”, “transport” and “stress” the most represented. Transcription profiles revealed considerable differences in regulation of major phytohormone signalling pathways: whereas Salicylic and Abscisic Acid pathways were up-regulated in the resistant genotype, Jasmonate and Ethylene pathways were down-regulated in the susceptible one. As potential Resistance-genes we identified a mildew resistance locus O (MLO)-like gene, and MLO-related transcripts. Also, several pathogenesis-related genes were up-regulated in the resistant and exclusively down regulated in the susceptible genotype. Pathogen effectors identified in both inoculated libraries, as e.g. the rust Rtp1 transcript, may be responsible for the down-regulation of defence-related transcripts. The two genotypes contained 4,892 polymorphic contigs with SNPs unevenly distributed between different functional categories. Protein degradation (29.7%) and signalling receptor kinases (8.2%) were the most diverged, illustrating evolutionary adaptation of grass pea to the host/pathogens arms race.

**Conclusions:**

The vast array of novel, resistance-related genomic information we present here provides a highly valuable resource for future smart breeding approaches in this hitherto under-researched, valuable legume crop.

**Electronic supplementary material:**

The online version of this article (doi:10.1186/s12870-014-0376-2) contains supplementary material, which is available to authorized users.

## Background

Rusts are among the most important diseases of legumes [1] and grass pea (*Lathyrus sativus* L.) is not an exception [2-4]. Rusts are caused by biotrophic fungi that keep infected host cells alive for their development. They form elaborate intracellular accommodation structures called haustoria, which maintain an intimate contact between fungal and plant cells over a prolonged period of time [5].

Rust in *Lathyrus* spp. is caused by *Uromyces pisi* (Pers.) Wint and *U. viciae-fabae* (Pers.) J. Schröt [6,7], but and in addition to *Lathyrus*, *U. pisi* infects a broad range of other legumes too [7,8]. Plants have developed multifaceted defence responses, many of which are induced only upon pathogen attack. These responses may include induction of pathogenesis related (PR) genes, the production of secondary metabolites (as e.g. phytoalexins), as well as the reinforcement of cell walls [9]. Associated with these responses may be the production of reactive oxygen species (ROS) and the induction of localized cell death (the hypersensitive response, HR) [10]. The induction of this basal plant defence machinery occurs upon the recognition of conserved molecules which are present in a variety of microbial species, but absent in the host. These pathogen associated molecular patterns (PAMPs) are molecular components highly conserved within a class of microbes, where they have essential functions for their fitness or survival [11]. These include, for example, fungal chitin, β-glucan and ergosterol. The specific virulence factors of the pathogen, known as fungal effectors, are recognized by corresponding resistance (R) genes of the host plant. Both rust-causing pathogens of *Lathyrus* are able to efficiently overcome R-gene based resistance [12]. To date, most fungal effectors identified are lineage-specific small secreted proteins (SSP) of unknown function [13,14]. The *U. viciae-fabae* rust transferred protein 1 (Rtp1) was the first fungal effector visualized in the host cytoplasm and nucleus after *in planta* secretion by the rust fungus [15]. Rtp1 belongs to a family of cysteine protease inhibitors that are conserved in the rust species (order *Pucciniales* formerly known as *Uredinales*) [16].

Gene-for-gene resistance is associated with the activation of, for instance, the salicylic acid (SA)-dependent signalling pathway, leading to expression of defence-related genes like PR1, the production of ROS and finally to programmed cell death [17,18]. Other phytohormones involved in plant/pathogen interaction are ethylene (ET) and jasmonates (JA). Plant defence responses appear specifically adapted to the attacking pathogen, with SA-dependent defences acting mainly against biotrophs, and JA- and ET-dependent responses acting mainly against necrotrophs [5,19,20].

Grass pea is a diploid species (2n = 14) with a genome size of approx. 8.2 Gbp [21]. Although grass pea is primarily self-pollinated, a 2 to 36% outcrossing rate was reported, depending on location and genotype [22-24]. Outcrossing is mainly driven by pollinators, and therefore can be minimized when grown in isolation [22]. There is a great potential for the expansion of grass pea in dry areas and zones that are becoming more drought-prone as a result of climate change [25]. Partial resistance to *U. pisi* has been reported in grass pea as a clear example of prehaustorial resistance, with no associated necrosis. This resistance is due to restriction of haustoria formation accompanied by frequent early abortion of the colonies, reduction in the number of haustoria per colony and decreased intercellular growth of infecting hyphae [26]. Though prehaustorial resistance is typical for non-hosts, it has also been implicated in host partial resistance [27,28] and is common in resistance of major cool season grain legumes against rusts [1,29]. Additionally, resistant *Lathyrus* genotypes may serve as a source of new and useful genetic traits in the breeding of related major legume crops such as peas, lentils and vetches. Cross-incompatibility has been reported between pea and *L. sativus*, but successful fusion of *Pisum sativum* and *L. sativus* protoplasts [30] creates new possibilities for gene transfer between these species. However, the slow progress in understanding the genetic control of important traits, such as disease resistance, in *Lathyrus* species hampered the development of modern cultivars or the introgression of their interesting traits into related species.

In economically important warm season legumes such as common bean and soybean, complete monogenically controlled resistances to rusts and associated rust resistance genes have been described together with closely linked markers for use in marker assisted backcrossing [29,31-34]. By contrast, most rust resistances described so far in cool season food legumes are incomplete in nature and the genetic basis of resistance is largely unknown. Although QTL mapping studies confirmed the polygenic control of resistance as e.g. in pea [35], faba bean [36] and chickpea [37], no markers suitable for marker assisted selection (MAS) are available yet.

Genomic resources for grass pea are still scarce (e.g. in April 2014 the NCBI database contained only 178 EST sequences from *L. sativus* [38]), and the two linkage maps existing for grass pea do not contain sufficiently informative markers to bridge between them [4].

The advent of next-generation sequencing (NGS) technologies was an important breakthrough enabling the sensitive and quantitative high-throughput transcriptome analysis referred to as RNA-seq [39,40]. RNA-seq discriminated between microbial and host transcriptomes, during plant-microbe interactions, using original or phylogenetically related genomes as a reference for transcript annotation [41-44]. RNA-seq gene expression patterns provided also information on complex regulatory networks and on variations in expressed genes, such as SNPs and SSRs, in an increasing number of non-model plants [45] and thus may be well suited to overcome the bottleneck of lacking genomic resources in *Lathyrus*.

Here we employed RNA-seq to study the response of *L. sativus* to *U. pisi* infection. We used MapMan and metabolic pathway analyses to interpret the results and assessed allelic diversity in transcripts as a source for genic markers for future (comparative) mapping studies. In addition, the expression of a set of selected genes was measured by qRT-PCR to validate the RNA-seq results.

To our knowledge, this is the first study on the global expression profiling of genes in grass pea/pathogen interaction using NGS. Our results will assist the elucidation of pathways and genes associated with resistance to rust in grass pea and related species. This approach may represent one of the initial steps towards the development of effective strategies for resistance breeding against such a quickly evolving pathogen.

## Results

### Contigs from the RNA-seq transcriptomes of resistant and susceptible *L. sativus* genotypes

The RNA-seq libraries from control and inoculated leafs from the resistant genotype BGE015746 were united prior to assembly to generate a comprehensive data set enabling the generation of contigs of maximum length. They included 46,994,629 reads which were assembled into 105,288 contigs, ranging in size from 150 to 13,929 bp, with a mean contig length of 544 bp. The respective united library from the susceptible genotype BGE024709 comprised 72,566,465 reads which assembled in 119,870 contigs, with a size range of 150 to 15,658 bp and a mean contig length of 524 bp.

A reference assembly using both genotypes and treatments assembled in 134,914 contigs, ranging in size from 150 to 13,916 bp, with a mean contig length of 501 bp. The mapping and quantification of both genotypes’ libraries to the reference assembly allowed the analysis of their differential expression in response to *U. pisi* infection. 9,501 contigs were unique to the resistant and 15,645 contigs were unique to the susceptible genotype.

Redundancy of the reference assembly was checked using the clustering algorithm UCLUST, identifying only 49 (0.036%) transcripts with identity higher than 95%.

This Transcriptome Shotgun Assembly project has been deposited at DDBJ/EMBL/GenBank under the accession GBSS00000000. The version described in this paper is the first version, GBSS01000000.

### RNA-seq validation by quantitative RT-PCR assay

**Figure 1:**
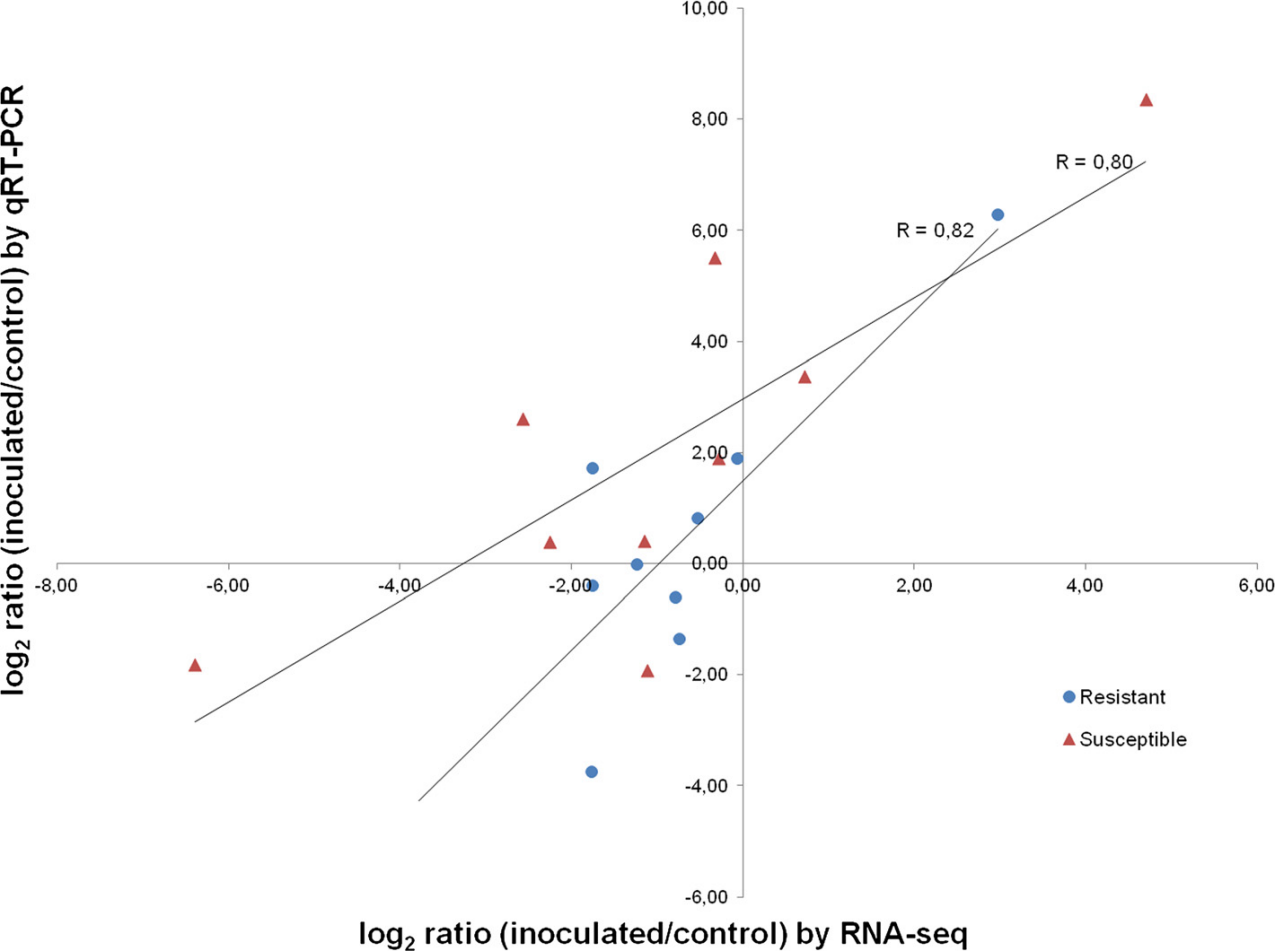
**Correlation between RNA-seq and qRT-PCR.** The relative expression levels obtained by RNA-seq using DEGseq and by qRT-PCR using the ΔΔCt method. Pearson’s correlation coefficient (R) between relative expression levels is shown above the trendline.

To validate the RNA-seq results, expression levels of a set of 9 selected genes were analysed by qRT-PCR. Genes were selected by their level of expression and transcript count, in order to represent a broad range of expression profiles. Further, the number of their transcripts differed between inoculated and control samples by log2 ratios ranging from −6.39 to 4.70 at q-values < 0.05. Their read count numbers were generally higher than 100, with exception of contig a45744;151, “mitochondrial chaperone BCS1”, with 2 counts in the resistant control and 36 counts in the resistant inoculated line, and contig a32859;123 “seed maturation protein”, with 3 counts in the susceptible inoculated line and 104 counts in the resistant inoculated line (Table 1). The best housekeeping genes for normalization suggested by the geNorm software were, for the resistant genotype samples, “β-tubulin” (a6507;507) and “photosystem I P700 apoprotein A2” (a160;902), and “O-methyltransferase” (a5102;390), for the susceptible genotype. A good correlation (R = 0.82 for the resistant and R = 0.80 for the susceptible genotypes) was observed between the log2 fold changes measured by RNA-seq and qRT-PCR (Figure 1).

**Table 1:** **Log2 fold expression results for RNA-seq and qRT-PCR experiments**

**Reference assembly contig**	**BLAST hit**	**BGE015746**							**BGE024709**						
		**Control counts**	**Control RPKM**	**Inoculated counts**	**Inoculated RPKM**	**Inoculated/control DEGSeq (log2)**	**DEGSeq q-value**	**Inoculated/control qPCR (log2)**	**Control counts**	**Control RPKM**	**Inoculated counts**	**Inoculated RPKM**	**Inoculated/ control DEGSeq (log2)**	**DEGSeq q-value**	**Inoculated/ control qPCR (log2)**
a1310;251	Chromodomain helicase DNA-binding protein	4063	0.0362	6436	0.0396	−0.53	1.0E-77	0.83	4149	0.0287	8009	0.0379	−0.33	1.7E-33	5.51
a15017;192	Type IIB calcium ATPase	850	0.0135	1855	0.0204	−0.07	3.2E-01	1.90	1165	0.0144	4635	0.0391	0.72	6.5E-64	3.38
a19532;154	Amino acid transporter	1965	0.0312	1328	0.0145	−1.76	8.4E-266	1.73	2045	0.0251	2224	0.0187	−1.15	3.2E-151	0.42
a22579;158	Hypothetical protein MTR 2 g062700	1329	0.0592	901	0.0277	−1.76	2.2E-179	−0.39	602	0.0208	245	0.0058	−2.57	3.0E-135	2.61
a2401;404	Lectin	776	0.0415	1061	0.0392	−0.75	1.5E-27	−1.35	813	0.0337	4283	0.1214	−1.12	6.8E-125	−1.92
a32859;123	Seed maturation protein	276	0.0438	185	0.0202	−1.77	1.9E-38	−3.74	104	0.0128	3	0.0003	−6.39	4.9E-39	−1.81
a45744;151	Mitochondrial chaperone BCS1	2	0.0004	36	0.0051	2.97	7.8E-05	6.29	7	0.0011	440	0.0480	4.70	1.2E-65	8.35
a5330;269	Alpha-galactosidase 1	1092	0.0373	1443	0.0341	−0.79	8.9E-43	−0.60	1203	0.0319	2387	0.0433	−0.29	2.8E-08	1.91
a6560;334	GDSL esterase/lipase EXL3	1981	0.0904	1917	0.0604	−1.24	3.3E-160	0.00	2164	0.0765	1101	0.0266	−2.25	0.0E + 00	0.39

RPKM: reads per kilobase per million.

### Differential gene expression in resistant and susceptible *L. sativus* genotypes during infection

Differentially expressed contigs were grouped by expression patterns based on up- or down-regulation (log2 ≥ 2 or log2 ≤ −2; respectively, q-value ≤ 0.05) after inoculation. Within each expression pattern group, comparisons were performed between genotypes. Expression patterns were grouped in eight response types, according to their up- or down-regulation, in susceptible and resistant genotypes, respectively. The number of contigs and description of each group is summarized in Table 2. Most representative groups are group F (contigs down regulated in both genotypes) and H (contigs down-regulated only in the resistant genotype) with 2,516 and 1,606 contigs respectively, followed by group A that includes 814 contigs up-regulated in both resistant and susceptible genotypes upon infection. A detailed list with all the identified contigs, their description and expression pattern groups can be found in Additional file 1.

**Table 2:** **Classification of contigs according to their differential expression in the susceptible and resistant genotype upon infection with** ***U. pisi***

**Expression pattern group**	**Feature**	**# of contigs**
**A**	Up-regulated in Resistant	814
	Up-regulated in Susceptible	
**B**	Up-regulated in Resistant	32
	Up-regulated in Susceptible, higher in Susceptible	
**C**	Up-regulated in Resistant, higher in Resistant	56
	Up-regulated in Susceptible	
**D**	Up-regulated in Susceptible	319
**E**	Up-regulated in Resistant	576
**F**	Down-regulated in Resistant	2,516
	Down-regulated in Susceptible	
**G**	Down-regulated in Susceptible	548
**H**	Down-regulated in Resistant	1,606
**Total**		134,914

Up regulated: (log2 > = 2; q-value ≤ 0.05); Down-regulated: (log2 ≤ −2; q-value ≤ 0.05); higher in Susceptible: (log2 fold change between all resistant and susceptible genotype contigs < = −2; q-value ≤ 0.05); higher in Resistant: (log2 fold change between all resistant and susceptible genotype contigs > = 2; q-value ≤ 0.05).

**Figure 2:**
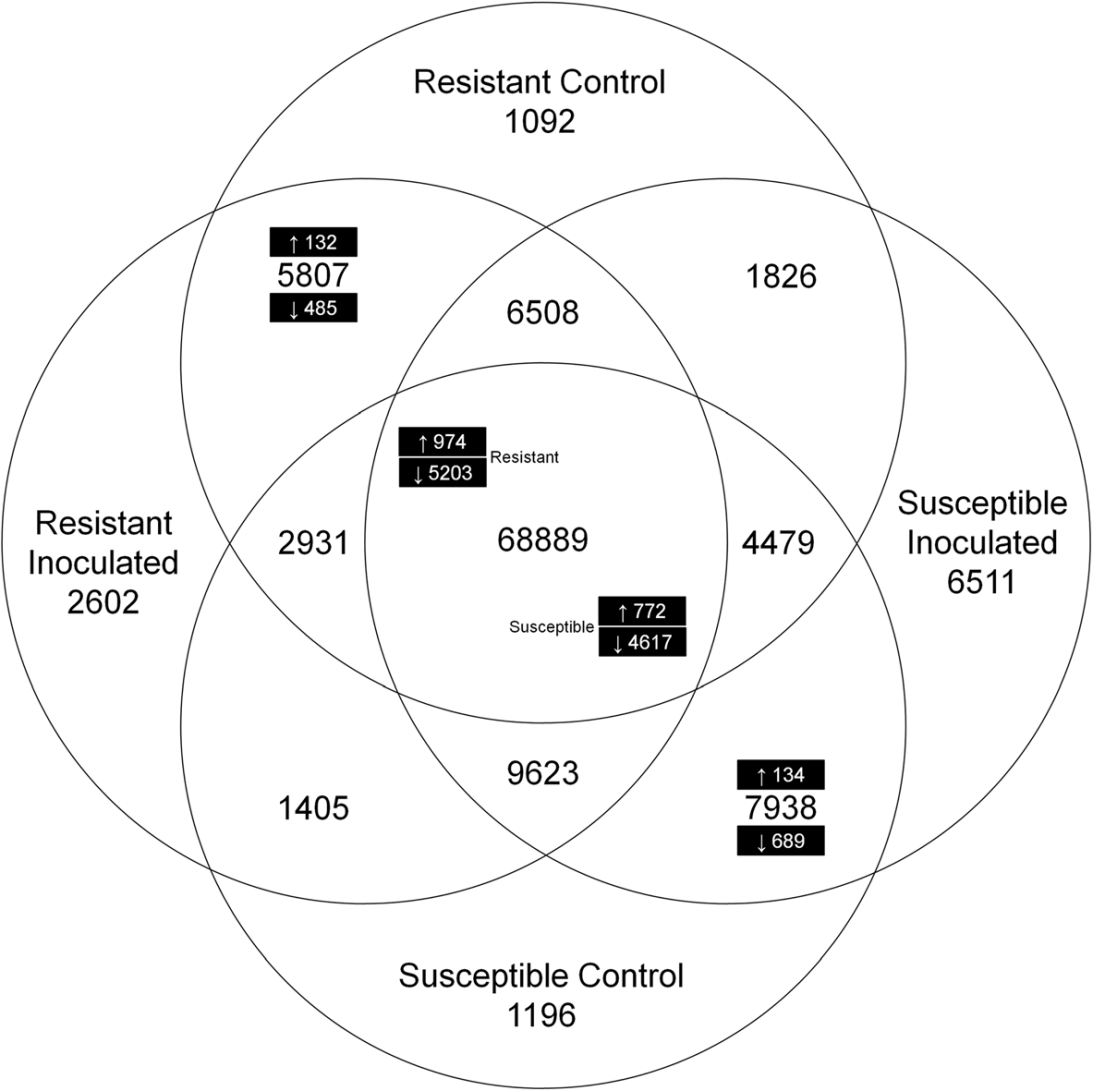
**Venn diagram of the number of unique and shared contigs between the two genotypes and its expression.** In black boxes the number of up (log_2_ fold ≥ 2) and down (log_2_ fold ≤ −2) regulated contigs in the inoculated condition versus control. Resistant genotype: BGE015746, susceptible genotype: BGE024709.

As depicted in Figure 2, from the 134,914 contigs that could be identified and quantified, 68,889 were shared among all libraries. Of these, 974 contigs were up-regulated and 5,203 contigs down-regulated in the resistant genotype BGE015746 and 772 contigs up- and 4,617 down-regulated in the susceptible genotype BGE024709. Furthermore, from the 5,807 contigs only present in the resistant genotype’s libraries, 132 were up- and 485 down-regulated (inoculated vs. control). From the 7,938 contigs only found in the susceptible genotype’s libraries, 134 were up- and 689 down-regulated.

### Annotation

From the 134,914 contigs detected in all libraries, 50,937 (37.75%) contigs could be matched via BLAST to entries in plant databases and 961 (0.71%) matched only to fungal databases. The latter contigs were present only in the inoculated libraries. Also, 4,558 contigs were absent in control samples and found exclusively in fungal databases, or with a higher bit-score in fungal databases than in plant databases and thus, most probably correspond to *U. pisi* sequences.

**Figure 3:**
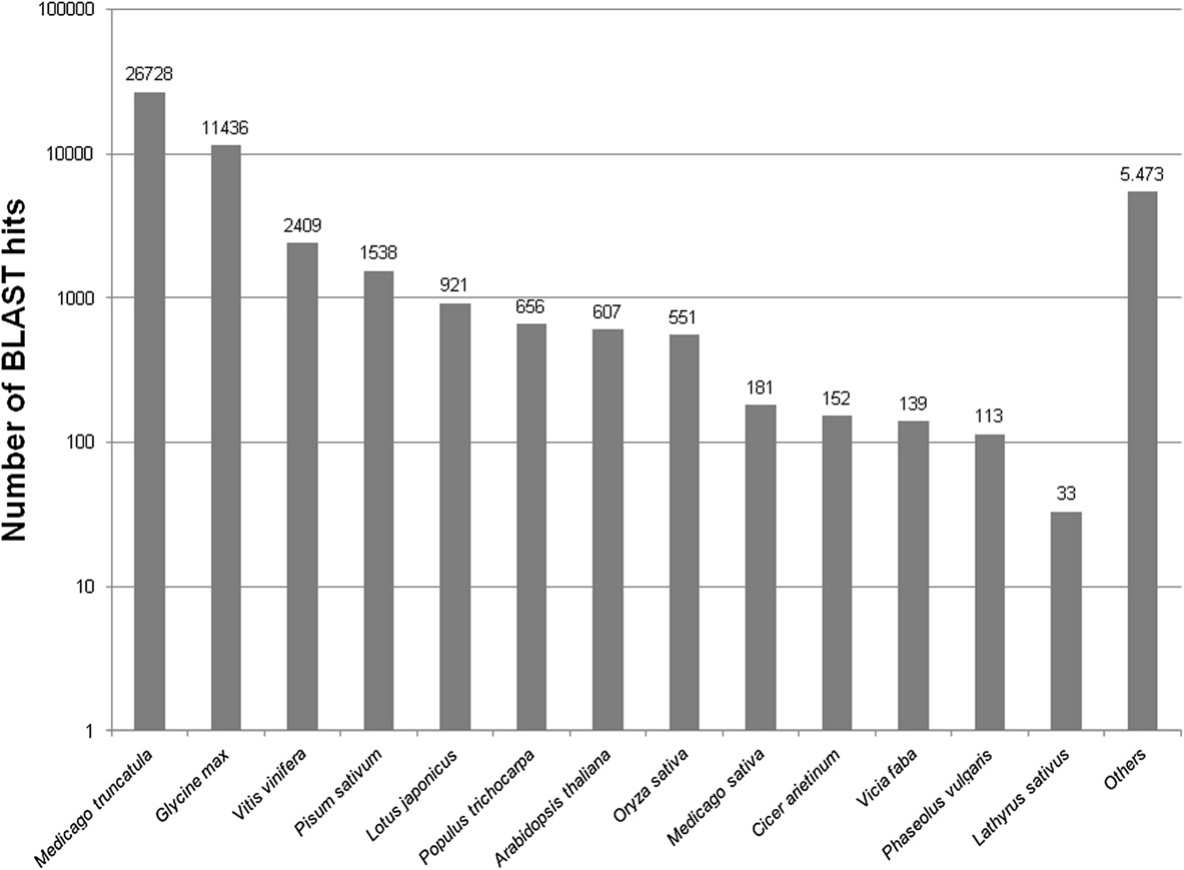
**Number of contigs that could be BLASTed to different plant species.**

As indicated in Figure 3, BLAST produced hits mainly to other legume species with frequencies in the order *Medicago truncatula* (26,728; 19.81%), *Glycine max* (11,436; 8.48%), *P. sativum* (1,538; 1.14%) and *Lotus japonicus* (921; 0.68%). *Vitis vinifera* (2,409; 1.79%), *Populus trichocarpa* (656; 0.49%) and the model *Arabidopsis thaliana* (607; 0.45%) were the best matching non-legume species. BLAST hits from *L. sativus* comprised only 0.02% (33 contigs) of the total illustrating the scarcity of *Lathyrus* entries in the data bases.

From the 4,558 contigs that were absent in control samples and found exclusively in fungal databases, or with a higher bit-score in fungal databases, 20 contigs from the 49 accessions described in UniProtKB/Swiss-Prot and UniProtKB/TrEMBL as *U. viciae-fabae*, were identified (see list in Additional file 2). None of these 20 contigs were significantly differentially expressed between the two inoculated genotypes. For example, among the eight contigs out of the 20 without a plant database hit, five were homologous to “invertase 1”, and the three others to “rust transferred protein – Rtp1”, “amino acid transporter” and “putative permease”. Six other contigs absent in control samples and found exclusively in fungal databases or with a higher bit-score in fungal databases, were homologous to housekeeping genes that can be found throughout different kingdoms (three “tubulin beta chain”, two “succinate dehydrogenase” and one “plasma membrane (H+) ATPase”.

**Figure 4:**
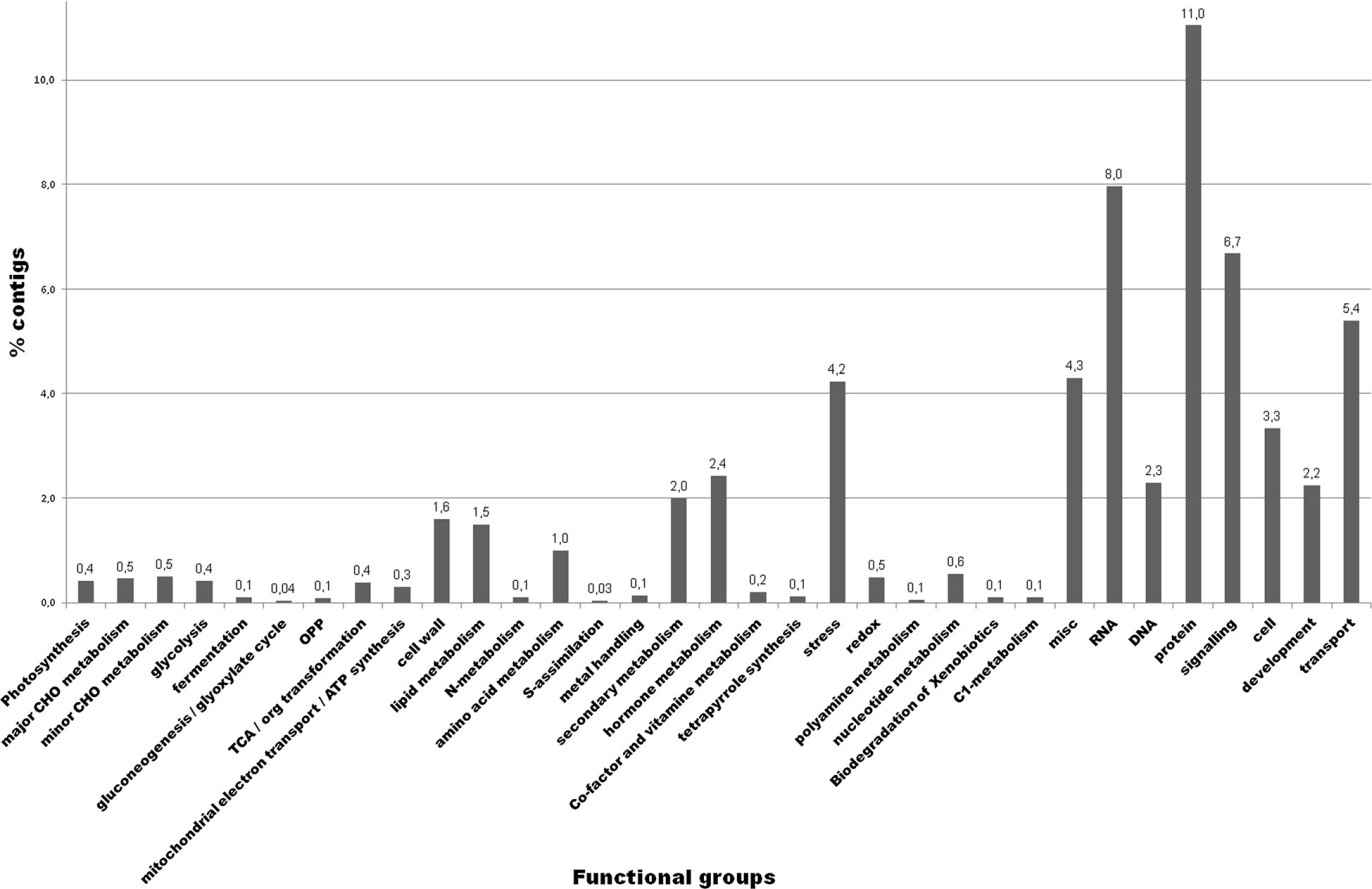
**Percentage of contigs assigned in each main functional category.**

Functional annotation of the contigs via Mercator and MapMan, depicted in Figure 4, grouped 60.4% of them into 33 main functional categories, of which the categories “protein” (11.0%), “RNA” (8.0%), “signalling” (6.7%), “transport” (5.4%) and “stress” (4.2%) were most crowded. A total of 39.4% could not be assigned to any functional category.

**Figure 5:**
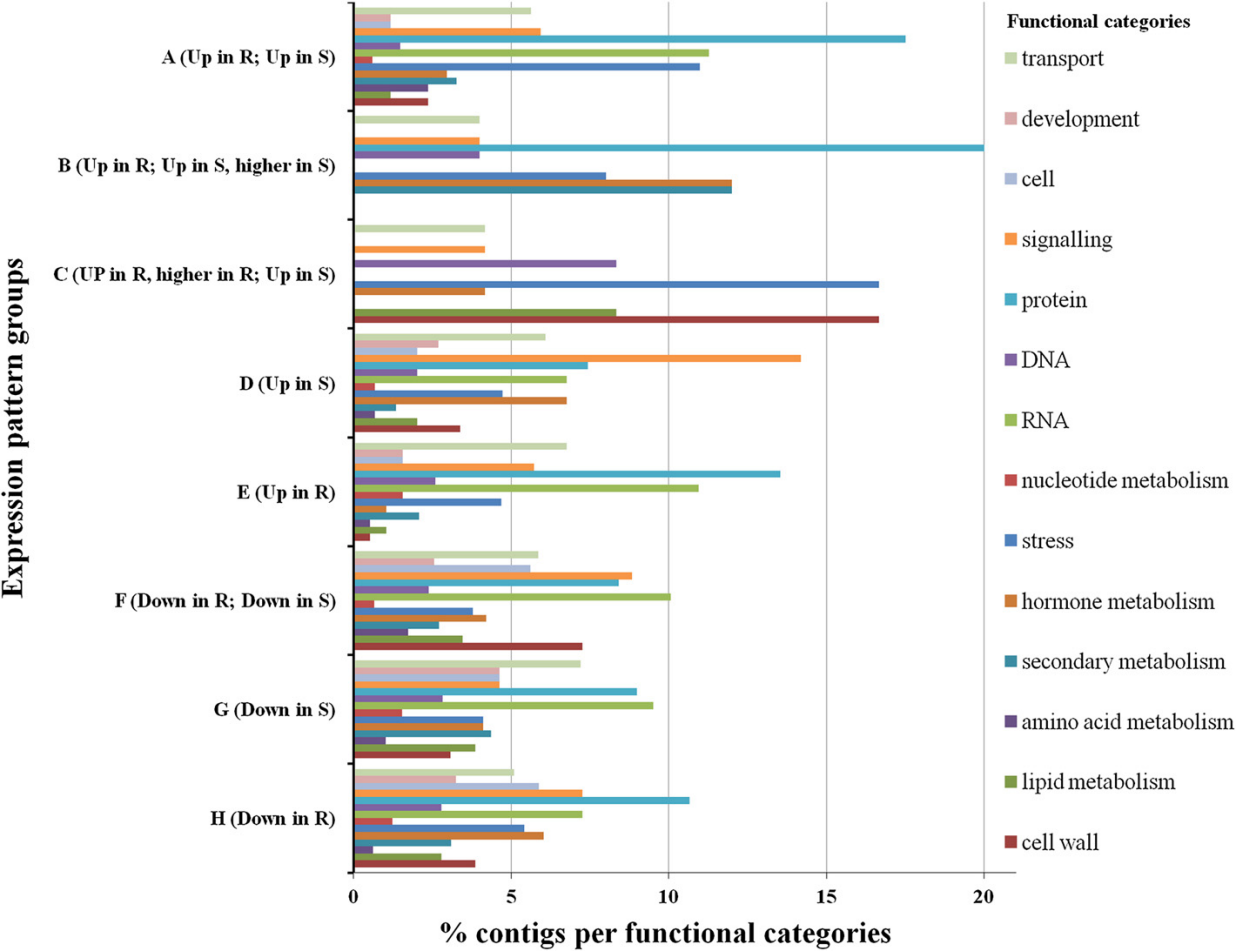
**Percentage of contigs assigned in each functional category for each expression pattern group. A** - contigs up-regulated in both resistant and susceptible genotypes similarly; **B** - contigs up-regulated in both genotypes, with a higher expression in the susceptible genotype; **C** - contigs up-regulated in both genotypes, with a higher expression in the resistant genotype; **D** - contigs up-regulated only in the susceptible genotype; **E** - contigs up-regulated only in the resistant genotype; **F** - contigs down-regulated in both resistant and susceptible genotypes similarly; **G** - contigs down-regulated only in the susceptible genotype; **H** - contigs down-regulated only in the resistant genotype.

Analysis of functional categories, within each expression pattern group, identified differences among the functions present within each group. Comparisons were also performed among the different expression profiles in each category (Figure 5). Transcripts included in the functional categories “stress” and “protein” were present at a higher percentage in up-regulated expression pattern groups (“stress” in A, B and C; “protein” in A, B and E), while the functional category “cell” was present at higher percentage in down-regulated expression pattern groups (F, G and H). The most prominent functional category in group C contigs up-regulated in both genotypes, with a higher expression in the resistant genotype was “cell wall”. “Lipid metabolism” and “DNA” were also over-represented. However, also the down-regulated groups F, G, and H contained a considerable number of contigs from the “cell wall” category. In group B, joining contigs up-regulated in both genotypes with a higher expression in the susceptible genotype, the categories “secondary metabolism” and “hormone metabolism” were over-represented. Interestingly, the functional category “signalling” was over-represented in contigs up- regulated only in the susceptible genotype, as in group D.

### Biotic stress related proteins

In order to restrict the number of analysed contigs to the ones probably more directly related to resistance, we focused mostly on contigs up-regulated at a higher ratio, or exclusively, in the resistant genotype (groups C and E), contigs exclusively down-regulated in the susceptible genotype (group G) and contigs exclusively down-regulated in the resistant genotype (group H).

From the subcategory “stress.biotic”, two contigs in group E corresponded to the well- studied mildew resistance locus O (MLO) gene which was first identified in barley, conferring resistance to powdery mildew [46]. Also, from a total of 25 “MLO-like” contigs, 12 were differentially expressed. One of these was down-regulated in the resistant genotype (group H). These might be related to MLO susceptibility genes, as reported by several previous studies [47-49]. Interestingly, in the susceptible genotype, one “PREDICTED: beta glucosidase 12-like”, identified by Mercator as “PENETRATION 2”, required for MLO-mediated resistance and belonging to the functional category “secondary metabolism”, was down-regulated (group G). Group G also contained one “acidic endochitinase” and two LRR proteins, one TIR-NBS-LRR and one containing LRR and NB-ARC domains. In group C, a pathogenesis related protein 1 (PR-1) contig was identified.

The subcategory “stress.abiotic” contained, i.a., genes involved in response to heat that also respond to biotic stresses. For example, in group C and E, we identified one “DNAJ heat shock protein” in both groups, three “heat shock protein 70 family” (group E) and one “18.1 kDa class 1 heat shock protein” (group E). Group G, however, contained one “DNAJ homolog subfamily B member” and one “double Clp-N motif-containing P-loop nucleoside triphosphate hydrolases superfamily protein”.

Several contigs related to secondary metabolism were exclusively up-regulated in the resistant genotype (group E). These comprised a “reticuline oxidase-like protein” involved in alkaloid biosynthesis, an “isoflavone 2’hydroxylase”, functioning in the isoflavonoid biosynthesis pathway, a “dihydroflavonol-4-reductase”, with roles in the flavonoid and brassinosteroid metabolic pathway and an “AMP-dependent CoA ligase”, acting in the JA and lignin biosynthesis pathways. In group G, 17 contigs were related to secondary metabolism including four involved in the flavonoid pathway, two in the isoprenoid/terpenoid pathway and one “WAX 2-like” involved in wax biosynthesis.

PTI (pathogen-associated molecular pattern triggered immunity) relies on an efficient signalling network in order to control the infection [50]. Receptor kinases are important for the plant’s pathogen recognition and their expression may be constitutively expressed or up-regulated in resistant genotypes or down-regulated in susceptible genotypes in response to effectors from the pathogen. Receptor kinases and kinases exclusively up-regulated in the resistant genotype and contained in group E may be part of such signalling cascades. These included one protein kinase with thaumatin (PR-5) domain, six “DUF 26”, one “CRINKLY4”, one “FERONIA receptor like kinase”, and also a MAP kinase “MAPKKK5” and a G-protein “zinc finger (Ran-binding) family protein”. In contrast, the down-regulation of such transcripts in the susceptible genotype (group G) may contribute to susceptibility. Here we identified three “DUF 26”, three LRR (“NIK1”, “RKF1” and “PXY”), two G-proteins (“guanine nucleotide-binding protein” and “dynamin-related protein 1E-like”), two MAP kinases (“PAS domain-containing protein tyrosine kinase family protein”) and three genes involved in calcium signalling (“calcium-transporting ATPase”, “calmodulin-binding heat-shock protein” and “calmodulin-domain protein kinase 9”). Interestingly, calmodulin also plays a role in the MLO response, where the lack of a calmodulin binding site decreases its defence response [51].

The “cell wall” category contained seven cellulose synthase contigs: one in group E “IRREGULAR XYLEM 3 (IRX3)” and four in group C (three “IRX1” and one “CESA1”). In group G, we identified two cellulose synthase “IRX14” and two “pectinesterase inhibitor” contigs.

From the genes normally associated with defence response, only one “endo-beta-1 3-glucanase” was identified in group C, while two others “endo-beta-1 3-glucanase” were detected in group G. Also in group G, we identified two “peroxidase” and two “glutathione S-transferase” genes.

### SNPs in resistance pathways

**Figure 6:**
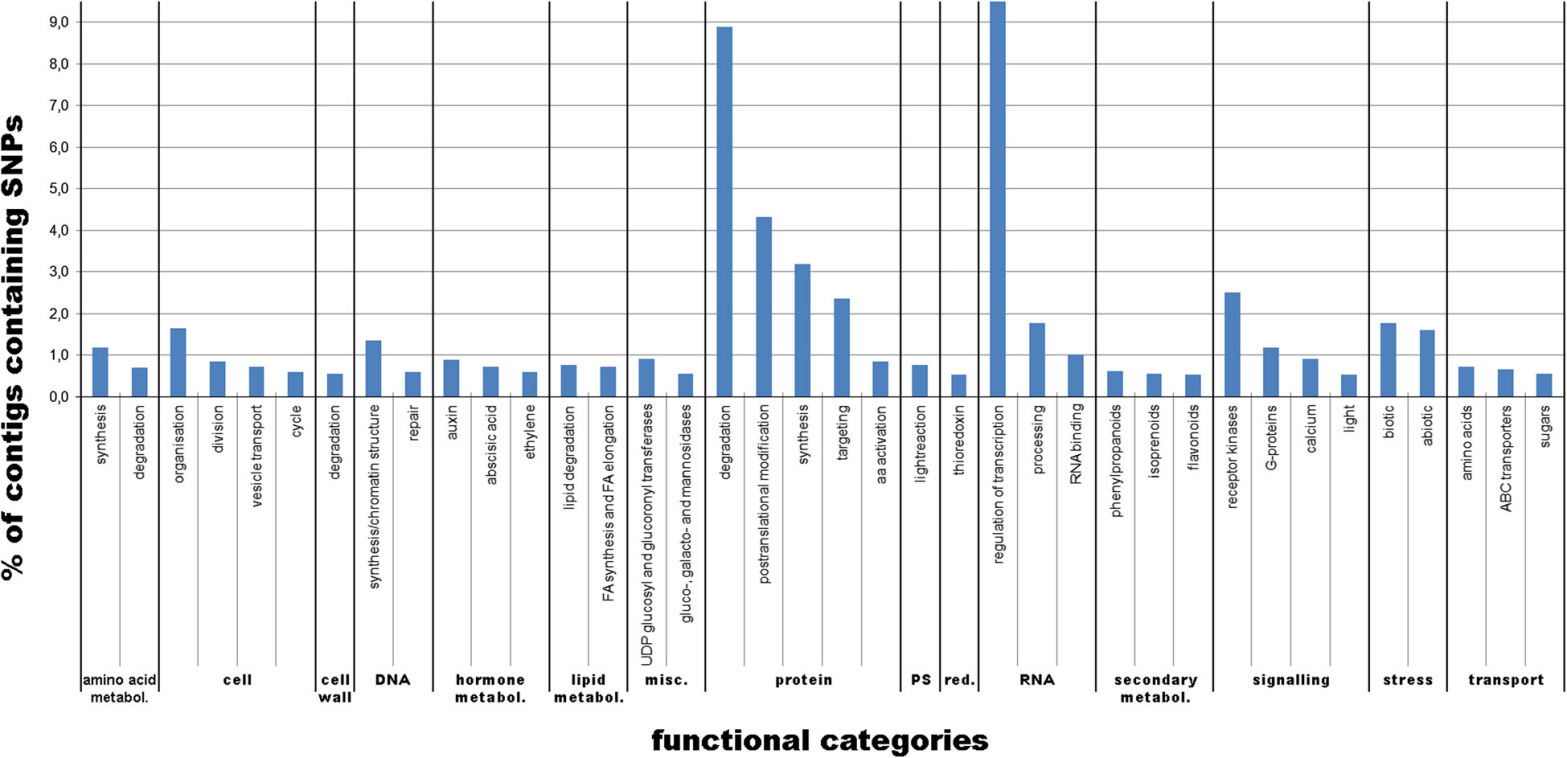
**Percentage of contigs containing SNPs between the resistant and susceptible genotypes in each Mercator mapping functional sub-category.** FA: fatty acid; metabol: metabolism; misc.: miscellaneous; PS: photosynthesis; red.: redox.

In the 68,889 contigs present in both the susceptible and the resistant genotypes, we identified 2,634 contigs containing Single Nucleotide Polymorphisms (SNPs) discriminating between their respective alleles. The number of SNPs in functional (MapMan) categories varied considerably. The categories “RNA regulation of transcription” (9.5%) and “protein.degradation” (8.9%) contained by far the most SNPs, followed by the protein-related categories “protein.postranslational modification” (4.3%) and “protein.synthesis” (3.2%). Other categories including the most SNP-containing contigs were “signalling.receptor kinases” (2.5%), “protein.targeting” (2.4%) and the stress related categories, “stress.biotic” (1.8%) and “stress.abiotic” (1.6%) (Figure 6).

### EST-SRR development

200 EST-SSR potential polymorphic markers between the two genotypes were designed. EST-SSRs were identified by the Phobos software [52], using as search parameters, perfect SSRs with a repeat unit lenght of two to six nucleotides. Polymorphisms between the resistant and susceptible genotypes were manually identified and flanked by primer pair using the Primer3 software [53]. To validate the EST-SSR sequences, 40 primer pairs were randomly selected for PCR amplification to confirm the presence of size polymorphism between the two accessions. PCR reactions were conducted twice in order to confirm the results. From the total 40 EST-SSR tested, 25 (62.5%) primer pairs successfully amplified polymorphic fragments between the two accessions. 6 (15.0%) primer pairs amplified monomorphic fragments and 5 (12.5%) produce a very complex pattern. The remaining 4 (10.0%) primer pairs were not able to produce any fragments.

## Discussion

*Lathyrus* spp. is a potential source of resistance to several pathogens [4,25] and especially *L. sativus* provides resistance to several fungal and bacterial diseases [26,38,54,55]. However, the lack of genetic and/or genomic information was a barrier to further identify resistance-related genes and to use them in breeding.

In the present study we therefore attempted to improve this unfavourable situation by identifying ESTs and SNPs, potentially involved in resistance, that may be used in future smart breeding approaches. We describe for the first time a high-throughput transcriptome assembly of grass pea/pathogen interaction, using genotypes contrasting in response to rust infection, to unravel the involved partial resistance mechanism and associated resistant genes.

Our study has identified a large number of differentially expressed genes corresponding to biological categories that are thought to be most relevant in grass pea response to rust. A limitation of our study is the fact that only a single pooled sample was investigated for each genotype and condition. Although the biological variance could not be assessed in the bulked approach, the large number of individual samples in the pool is likely to level out many of possible outliers. Nevertheless, the validation of twelve genes by RT-qPCR, using three biological replicates, provided a good correlation with RNA-seq results.

Another motive that could also be influencing our results is that we used different cDNA synthesis primers, oligo(dT) for the RNA-seq and poly(A) for RT-qPCR, what might yield different quantities of poly-adenylated and non-adenylated transcripts.

Our study was severely hampered by the low number of annotated sequences, which is due to the lack of a reference genomic sequence for *Lathyrus*. Nevertheless, we could annotate between 34% and 46% of differentially expressed contigs to hits in plant databases, depending on the genotype and the infection status of the plants. We further developed new gene-based molecular tools as e.g. expressed sequence tags, gene-based simple sequence repeats (EST-SSR) and SNP-based markers. Moreover, we identified a number of *U. pisi* effectors in the infected tissues though the overall low number of observed fungal transcripts probably reflects the low quantity of fungal structures in early-infected leaves [56]. Thus, our present study will help to overcome the problems we encountered in previous work, where the transfer of molecular markers from close related species had a very low rate of success (18% for pea EST-SSRs and 6% for pea genomic SSRs, [57]) Therefore, the present RNA-seq libraries will boost the availability of specific EST-SSRs and SNP-based markers that will be equally important for future development of more effective grass pea resistance breeding approaches.

The high amplification rate of the developed EST-SSRs validates the quality of the RNA-seq data. The few primers that failed to produce amplification products or produced amplicons with an unexpected pattern may be caused by the location of the respective primers across splice regions or the presence of a large intron, since genomic regions are absent from cDNA. In addition also primers could be derived from chimeric cDNA clones [58].

Besides the novel markers, the deep insights into pathogenesis-related mechanisms provided by this study are of particular interest. The most interesting pathogenesis-related protein that we identified, the “MLO-like protein” is involved in signalling in response to biotic stress. MLO was described for the first time in barley, where it conferred partial resistance to powdery mildew by inducing the thickening of the cell wall at fungal penetration sites [46]. Two “MLO-like” contigs were up-regulated exclusively in the resistant genotype (group E), and perhaps related to this, also in group E and more strongly up-regulated in the resistant genotype (group C), we identified cellulose biosynthesis genes. The exclusively resistance-up-regulated group E contained one “IRREGULAR XYLEM 3” (IRX3) gene and three “IRX1”. Additionally, one “cellulose synthase 1” (CESA1) was stronger up-regulated in the resistant genotype than in the susceptible one (group C). Consistent with the assumed importance of MLO signalling for rust resistance, some genes important for MLO function as e.g. “calmodulin”, involved in calcium signalling as a prerequisite for MLO function [51], were down-regulated in the susceptible genotype (group G). Another gene involved in MLO resistance and down-regulated in the susceptible genotype, is the glycosyl hydrolase “PENETRATION 2” gene [59]. Since rust resistance in grass pea is of prehaustorial type we consider MLO as a candidate R-gene. In order to confirm this assumption, callose deposition, as a potentially durable resistance mechanism against rusts, should be further investigated in rust-resistant and susceptible grass pea genotypes. The down-regulation of several “MLO-like” contigs in response to infection in both, susceptible and resistant genotypes, does not necessarily contradict our assumption, since several MLO orthologs were demonstrated to function as susceptibility genes [47-49].

Plant responses to biotic stressors are, i.a., controlled by phytohormones as e.g. salicylic acid (SA), abscisic acid (ABA), jasmonates (JA) and ethylene (ET). Differences in expression of hormone-related genes of the susceptible and resistant genotype, in response to the pathogen, also occurred in our gene expression patterns. For example, plant resistance to biotrophic pathogens is mainly controlled by the SA pathway [18] and the importance of SA in the induction of systemic acquired resistance in legumes against rust fungi has been reported [60,61]. In our study an inducer of the SA pathway, the “ethylene response factor 5” (ERF5) gene, which at the same time inhibits the JA and ET biosynthesis pathways [62], was exclusively up-regulated in the resistance genotype (group E), whereas two Apetala2/Ethylene Responsive Factor (AP2/ERF) transcription factor genes, important for the regulation of defence responses [63], were down- regulated in the susceptible genotype (group G).

ABA regulates defence responses through its effects on callose deposition and production of ROS intermediates [18], activating also stomata closure as a barrier against pathogen infection [64]. In the resistant genotype, the transcript for “9-cis-epoxycarotenoid dioxygenase 2”, a key regulator of ABA biosynthesis in response to drought [65], and involved in the crosstalk between ABA and SA signalling in plant-pathogen interactions [66], was up-regulated (group E), whereas several transcripts engaged in ABA, auxin and JA signalling, were down-regulated in the susceptible genotype (group G). This is consistent with a susceptible response to a biotroph attack [18].

The image emerging from the transcription profiles, of the resistant and susceptible genotype, further highlights that pathogenesis related (PR) proteins are key players in *Lathyrus*-rust interactions since several PR genes were mainly up-regulated in the resistant genotype, after inoculation. Among these were two chitinases (PR-3 and PR-9) involved in the degradation of the fungal cell wall [9] and a thaumatin (PR-5) gene, which causes an increase of the permeability of fungal membranes by pore-forming mechanisms [67]. In group E, we found a “pathogenesis related protein 1” (PR-1) and a “protein kinase-coding resistance protein”, a receptor kinase with a thaumatin domain (PR5K), presumably involved in thaumatin signaling and described previously as delaying infection [68]. Another important PR-gene, an “acidic endochitinase” (PR3), was down regulated exclusively in the susceptible genotype. Genes involved in secondary metabolism were also detected. Legumes utilize flavonoids, notably isoflavones and isoflavanones, for defence against pathogens and as signalling molecules, with a number of phenylpropanoids having antimicrobial activity and restricting pathogen growth and disease symptoms [69]. In group G, we identified a “reticuline oxidase-like protein”, up regulated in non- race-specific resistance to stripe rust in wheat [70], an “isoflavone 2′-hydroxylase” from the isoflavonoid pathway [71] and a “dihydroflavonol-4-reductase” catalysing the first enzymatic step in anthocyanin biosynthesis, in the flavonoid pathway [72].

Also, exclusively down regulated in the susceptible genotype, we found some genes important for defence response within the miscellaneous category, like “endo-beta-1 3-glucanase”, “glutathione S-transferase” (GST) and “peroxidase”. In plants, beta-glycosidases serve a number of diverse and important functions, including bioactivation of defence compounds, cell wall degradation in endosperm during seed germination, activation of phytohormones, and lignifications [73]. GSTs are detoxification-related proteins, protecting cells from secondary metabolites produced in response to pathogen attack, including peroxidases [74]. Finally, peroxidases function as resistance factors against invading fungi, inhibiting hyphal elongation, and when H_2_O_2_ is present, causing oxidative burst [75].

Effectors are expected to be excellent targets for the control of pathogens, but, unlike effectors from some other plant pathogens, relatively little is known about rust effectors [76]. In this study, several unigenes were identified in fungal databases. The most known rust effector identified was “rust transferred protein 1” (Rtp1). This effector aggregates into amyloid-like filaments in vitro [77]. Immunoelectron microscopy localized this effector to the extrahaustorial matrix protuberances extending into the host cytoplasm, although the exact role for this protein remains to be discovered [78]. Other *Uromyces* effectors identified in this study were “succinate dehydrogenase”, “invertase” and “permease”. From the total potential rust transcripts identified, a selection of effector proteins could be used as probes to identify the target host proteins as a first step in the development of effector-driven legume breeding, maximizing the durability of resistance against the quickly evolving rust pathogens [79].

From the DE contigs obtained in the present study, 2,634 presented SNPs between the resistant and the susceptible lines. The MapMan software aided in the functional categorisation of SNPs, revealing that the categories “RNA regulation of transcription” (9.5%) and “protein.degradation” (8.9%) contained by far the most SNPs. Within these categories, ubiquitins were most polymorphic (5.6%). Ubiquitins tag proteins for proteasome degradation and play a central role in signalling pathways [80]. Especially ubiquitin “E3 RING” and “SCF F-BOX” contigs contained a large number of SNPs (1.7% and 1.8% respectively). E3 RING and SCF F-BOX proteins are involved in several aspects of plant immunity ranging from pathogen recognition to both PTI to effector-triggered immunity (ETI). From the differentially expressed contigs identified as containing SNPs, we found one E3 RING, “PREDICTED: RING-H2 finger protein ATL2-like”, down regulated in the susceptible genotype. Four other functional categories, “RNA.regulation of transcription” (9.5%), “protein.postranslational modification” (4.3%), “protein.synthesis” (3.2%) and “signalling.receptor kinases” (2.5%), also contained significant numbers of polymorphisms. Especially the “signalling.receptor kinases” category may be of particular interest for further studies since receptor kinases recognize pathogen effectors and their rapid evolution, reflected by large numbers of polymorphisms, may represent plants adaptation to a rapidly changing spectrum of pathogens in the arms race between them and their hosts [81].

The large number of SNPs that we identified will be instrumental for the development of linkage and high-throughput association mapping approaches and for the expansion of our previous diversity studies in *Lathyrus* [57,82].

Our results provide an overview of gene expression profiles of contrasting *L. sativus* genotypes inoculated with rust, offering a valuable set of sequence data for candidate rust resistant gene discovery.

## Conclusions

Our transcriptome analysis provided comprehensive insight into the molecular mechanisms underlying prehaustorial rust resistance in *L. sativus*.

The differences in resistance between the two *L. sativus* genotypes investigated appear to be mainly due to the activation of the SA pathway and several pathogenesis related genes, including the ones regulated by MLO. The fastest-evolving pathways differentiating between the two genotypes are the general RNA’s regulation of transcription, followed by the Ubiquitin-26S proteasome system and having also as most mutated receptor-based signalling genes and biotic and abiotic stress related genes. The detected polymorphic SNPs will allow the development of new gene-based molecular tools. Altogether, 51 genes were identified as potential resistance genes, prioritizing them as specific targets for future functional studies on grass pea/rust interactions. Besides a plethora of pathogenesis-related host genes, 4,558 transcripts, including putative effectors, were also identified for the rust fungus *U. pisi*. As a consequence of the newly developed wider array of genetic and genomic resources, future work will focus on high throughput mapping of the genetic basis of disease resistance in *L. sativus* and eventual comparative mapping with other legume species, contributing all to an improved exploitation of this under used highly potential legume species.

## Methods

### Plant and fungal material, inoculation and RNA isolation

The two L*. sativus* genotypes, BGE015746 and BGE024709 analysed in the present work were kindly provided by the Plant Genetic Resources Centre (CRF-INIA), Madrid, Spain. Seeds were multiplied in insect proof cages in order to minimize outcrossing. Evaluation for their resistance against *U. pisi* demonstrated that BGE024709 is susceptible to rust, whereas BGE015746 displays partial resistance [26]. Upon infection, both genotypes present well-formed pustules, with no associated chlorosis or necrosis. They contrast, however, in disease severity (DS), i.e. the percentage of leaf area covered by the fungus. Whereas the partial resistant genotype has a DS = 9%, the susceptible one has a DS = 30%.

The *U. pisi* monosporic isolate UpCo-01 from the fungal collection of the Institute for Sustainable Agriculture-CSIC (Córdoba, Spain) was used for the experiment. Inoculum was multiplied on plants of the susceptible *P. sativum* cv. Messire before use.

Twenty-four plants per each genotype and treatment (inoculated/control) were used. Two-week-old *L. sativus* seedlings were inoculated by dusting all the plants at the same time with 2 mg of spores per plant, diluted in pure talk (1:10), with the help of a small manual dusting device in a complete random experiment. Inoculated and control plants were incubated for 24 h at 20°C, in complete darkness, and 100% relative humidity, then transferred to a growth chamber and kept at 20 ± 2°C under 14 h light (150 μmol m^−2^ s^−1^) and 10 h dark.

RNA was extracted from inoculated and non-inoculated fresh leaves collected 37 hours after inoculation. The material was immediately frozen in liquid nitrogen and stored at −80°C. RNA was isolated using the GeneJET Plant RNA Purification Mini Kit (Thermo Scientific, Vilnius, Lithuania), according to the manufacturer’s instructions. The extracted RNA was treated with Turbo DNase I (Ambion, Austin, TX, USA), and RNA quantification was carried out using the NanoDrop device (Thermo Scientific, Passau, Germany).

### Sequencing and quantification

For each of the 4 combinations, genotype and treatment (BGE015746 control, BGE015746 inoculated, BGE024709 control and BGE024709 inoculated) total RNA from 24 plants was extracted and pooled in equal amounts for sequencing. Three RNA-seq libraries (one for each genotype and one reference assembly, including all genotypes and treatments) were generated by GenXPro GmbH, Germany, using a proprietary protocol. In short, mRNA was captured from 20 μg of total RNA using Oligo dT(25) beads (Dynabeads; life Technologies). The purified mRNA was randomly fragmented in a Zn^2+^ solution to obtain approximately 250 bp long RNA fragments. cDNA was synthesized by reverse transcription starting from 6(N) random hexamer oligonucleotides followed by second strand synthesis. Barcoded Y-adapters were ligated to the cDNA and the library was amplified with 10 cycles of PCR. The libraries were sequenced on an Illumina Hiseq2000 machine. After Illumina paired-end sequencing, raw sequence reads were passed through quality filtering, thereby also removing sequencing adapter primers and cDNA synthesis primers. All high-quality reads were assembled using the Trinity RNA-Seq de novo assembly (Version: trinityrnaseq_r2011-11-26). In order to minimize the redundancy, CAP3 software [83] was also used with overlap length cutoff of 30 bp and overlap percent identity cutoff of 75%. Redundancy was tested using the clustering algorithm UCLUST ([84], available at http://drive5.com/usearch/manual/uclust_algo.html). The resulting contigs were annotated via BLASTX to publically available databases (ftp://ftp.ncbi.nlm.nih.gov/blast/db/FASTA/nr.gz, nr, plants only). To identify fungal transcripts, an additional BLASTX to public fungal databases (http://www.ebi.ac.uk/uniprot, UniProtKB/Swiss-Prot and UniProtKB/TrEMBL) was performed. The sequenced reads were mapped with novoalign software (V2.07.14; http://www.novocraft.com/) to the own assembled contigs. RPKM was calculated as the normalized transcript expression value [85]. Our obtained counts were subsequently passed through DEGSeq to calculate the differential gene expression (R package version 1.16.0) [86].

### SNP detection

SNPs were discovered between the two *L. sativus* genotypes using Joint-SNV-Mix [87]. The mappings from the transcriptome analysis were also analysed by Joint-SNV-Mix and the output was furthermore processed by GenXPro’s in-house software to detect SNPs discriminating the bulks. SNP calling was performed taking in account only the inoculated samples. A minimum coverage of 15 reads in each genotype in the inoculated condition was needed to call a SNP. Polymorphic contigs and their respective SNPs are listed in Additional file 3.

### EST-SSR development and genotyping

EST-SSRs were selected in silico by identifying the polymorphic SSRs between the two *L. sativus* genotypes. Identification of the SSRs was done using Phobos plug-in [52] for the Geneious software [88], using as search parameters, perfect SSRs with a repeat unit length of two to six nucleotides. Length polymorphisms were manually identified by aligning SSR-containing contigs of one genotype against the whole library of the other genotype. Primers were designed using Primer3 plug-in [53] for the Geneious software, using as parameters a melting temperature from 59 to 63°C, a GC content of 50 to 60% and a primer size raging from 18 to 24 nucleotides. The developed EST-SSR markers are listed in Additional file 4.

PCR reactions for the EST-SSRs genotyping were conducted using the M13 tail labelling strategy described by Schuelke [89] in a total volume of 10 μl containing 10 ng of template DNA, 0.04 μM of M13(−21) tagged forward primer, 0.16 μM of IRD700 or IRD 800 M13(−21) and 0.16 μM of reverse primer, 0.2 mM of each dNTP, 1.5 mM of MgCl_2_, and 0.2 unit of Taq DNA polymerase (Promega, Madison, USA). The amplification reaction consisted of a denaturing step of 5 min at 94°C, followed by 30 cycles of 30 s at 94°C, 45 s at 56°C, 45 s at 72°C, and 8 cycles of 30 s at 94°C, 45 s at 53°C, 45 s at 72°C. The reaction was terminated at 72°C for 10 min.

SSR fragments were resolved with 6.5% polyacrylamide gel using a LI-COR 4300 DNA Analyzer (Lincoln, NE, USA).

### Quantitative RT-PCR assay

1 μg of total RNA from each of three randomly chosen plants per genotype, per treatment (inoculated/control), was reverse transcribed in duplicates, using the High Capacity cDNA Reverse Transcription Kit (Applied Biosystems, Foster City, CA, USA) following manufacturer’s instructions. Three independent reverse-transcription reactions (RT) were performed for each cDNA sample in a total of nine samples per genotype, per treatment. For all genes studied, the product of each of these reactions was analysed in technical duplicates, in a total of six technical replicates per treatment. qRT-PCR reactions were performed with a iQ™5 Real-Time PCR Detection System (Bio-Rad, Munich, Germany). Primers were designed using the Primer3 software [53]. Primer sequences can be found in Additional file 5. For data analysis, the Genex software package (MultiD, Goteborg, Sweden), including the geNorm software [90] was used.

### Contig annotation and data analysis

In order to classify the contigs into functional categories, the Mercator pipeline for automated sequence annotation ([91], available at http://mapman.gabipd.org/web/guest/app/mercator) was employed. The mapping file was created excluding contigs without BLAST hit in previous analyses and accessing the following, manually curated databases: Arabidopsis TAIR proteins (release 10), SwissProt/UniProt Plant Proteins (PPAP), TIGR5 rice proteins (ORYZA), Clusters of orthologous eukaryotic genes database (KOG), Conserved domain database (CDD) and InterPro scan (IPR). The Mercator mapping file was then employed for pathway analysis by the MapMan software ([92], available at http://mapman.gabipd.org/web/guest/mapman).

Differentially expressed contigs were identified by comparing their expression in leaves of the resistant genotype BGE015746, control vs. inoculated, and of the susceptible BGE024709, control vs. inoculated, using DEGseq [86]. In cases where a particular transcript reacted in the same way in both genotypes, the total transcript count before and after inoculation was compared, allowing the identification of basal genotypic differences between the two genotypes.

### Availability of supporting data

The raw RNA-seq data supporting the result of this article is available in the Sequence Read Archive (SRA), with accession numbers SRS686331, SRS687370, SRS687371 and SRS687373. This Transcriptome Shotgun Assembly (TSA) project has been deposited at DDBJ/EMBL/GenBank under the accession GBSS00000000. The version described in this paper is the first version, GBSS01000000.

## Additional files

Additional file 1:
**Lists of genes in expression pattern groups mapped to the reference assembly.** RPKM: reads per kilobase per million. Additional file 2:
**List of contigs, mapped to the reference assembly, present only in the inoculated condition with a higher bit-score in fungal databases than in plant databases.**
Additional file 3:
**List of detected polymorphic SNPs between grass pea accessions BGE015746 and BGE024709.**
Additional file 4:
**List of detected polymorphic EST-SSRs between grass pea genotypes BGE015746 and BGE024709.**
Additional file 5:
**Contig information and primer sequences for qRT-PCR.**
